# Buckwheat Husk Biochars as Adsorbents for Cationic Dye Removal: Effect of Pyrolysis Temperature on Adsorption Performance

**DOI:** 10.3390/ma19142981

**Published:** 2026-07-10

**Authors:** Beata Doczekalska, Krzysztof Kuśmierek, Monika Bartkowiak, Andrzej Świątkowski

**Affiliations:** 1 Department of Chemical Wood Technology, Faculty of Forestry and Wood Technology, Poznan University of Life Sciences, 60-637 Poznan, Poland; monika.bartkowiak@up.poznan.pl; 2 Institute of Chemistry, Faculty of Advanced Technologies and Chemistry, Military University of Technology, 00-908 Warsaw, Poland; krzysztof.kusmierek@wat.edu.pl (K.K.); a.swiatkowski@wp.pl (A.Ś.)

**Keywords:** buckwheat husk, biochar, adsorption, Crystal Violet, Rhodamine B

## Abstract

Agricultural waste-derived biochars have recently attracted increasing attention as sustainable adsorbents for wastewater treatment. In this study, biochars (BHBs) produced from buckwheat husks at 500, 600, and 700 °C were investigated as novel adsorbents for the removal of the cationic dyes Crystal Violet (CV) and Rhodamine B (RhB) from aqueous solutions. The obtained materials were characterized using thermogravimetric analysis and surface functional group analysis to evaluate the influence of pyrolysis temperature on their physicochemical properties. The effects of initial adsorbent dose, solution pH, and ionic strength were assessed, while adsorption kinetics and equilibrium isotherms were analyzed to elucidate the adsorption mechanisms. It was found that the adsorption of both dyes depended on pH. CV adsorption was lowest in an acidic environment and increased with increasing pH from 3 to 9. RhB was most effectively adsorbed in an acidic environment. Its adsorption decreased as the pH increased from 3 to around 5, after which it stabilized. The adsorption of CV decreased with increasing ionic strength of the solution, whereas the adsorption efficiency of RhB remained unaffected. The adsorption kinetics of CV and RhB on BHBs were found to follow a pseudo-second-order mechanism controlled by film diffusion. The Langmuir, Freundlich, and Temkin models all provided good fits to the equilibrium experiments. The adsorption capacities of BHBs for CV and RhB decreased with increasing pyrolysis temperature and surface alkalinity of the biochars (BHB700 < BHB600 < BHB500). The adsorption capacities of biochars ranged from 41.00 mg/g (BHB700) to 56.10 mg/g (BHB500) for CV and from 9.74 mg/g (BHB700) to 13.24 mg/g (BHB500) for RhB. The study highlights the potential of buckwheat husk-derived biochars as sustainable adsorbents for the treatment of dye-contaminated wastewater and provides insight into the relationship between pyrolysis conditions and adsorption performance.

## 1. Introduction

Buckwheat (*Fagopyrum* Mill.) is an annual plant from the *Polygonaceae* Juss. family, classified as a pseudocereal, whose popularity is constantly growing worldwide due to its high nutritional and health-promoting properties [1]. Buckwheat grain is a rich source of vitamins (especially B vitamins), minerals (Zn, Mn, Cu, K, Na, Ca, Mg), and valuable antioxidant compounds such as rutin and catechins [1,2]. The main producers of buckwheat grain in the world are China, Russia, and Ukraine, closely followed by Kazakhstan and Poland, which ranks fifth in the global ranking [1,3]. The industrial technological process of obtaining buckwheat groats generates by-products. The most important of these are buckwheat hulls, constituting approximately 25–30% of the total mass of the processed raw material. The global annual production of buckwheat hulls is estimated at approximately 450,000 tons [3]. Although this biomass is traditionally used as an additive to animal feed or as a fuel, its low acquisition cost, abundance, and chemical structure make it an attractive raw material for advanced applications in environmental engineering.

Nowadays, rational processing of agricultural biomass waste (ABW) is the foundation of the circular bioeconomy and allows for the effective implementation of the United Nations Sustainable Development Goals (SDGs). This conversion contributes to the reduction in greenhouse gas emissions that would otherwise be generated during natural microbiological decomposition or uncontrolled burning of waste on agricultural fields [4]. Forecasts indicate that in Asian countries alone, ABW production will soon reach 4–5 kg per capita per month, which poses a serious ecological threat if effective disposal methods are not implemented [4]. One of the most promising and sustainable methods of managing waste biomass is its thermochemical conversion into biochar. Biochar, a carbon-rich and porous solid material, has gained significant importance in recent years in the context of supporting sustainable development and environmental protection [5,6]. Although it is traditionally associated with the process of biomass pyrolysis under oxygen-limited conditions, it can now be obtained using various thermochemical technologies, such as hydrothermal carbonization (HTC) [7], gasification [8], torrefaction [9], flash carbonization [10], hydrothermal liquefaction (HTL) [11], solvothermal liquefaction (STL) [12] and microwave pyrolysis [13].

The range of biochar applications now extends far beyond the traditional use of improving soil properties and increasing their water retention capacity [14]. It includes, among others, the remediation of areas contaminated with heavy metals [15,16,17], the removal of dyes and pharmaceuticals from wastewater [18,19], and carbon sequestration to mitigate climate change [6]. Furthermore, in the energy sector, biochar is used as a catalyst carrier in biodiesel synthesis [20] and as an electrode material in supercapacitors and hydrogen storage systems [21,22], providing an economical and more sustainable alternative to materials derived from fossil fuels.

In the face of increasing anthropogenic pollution of water resources worldwide, a key challenge is the search for cheap, efficient, and ecologically sustainable methods for removing toxic substances, of which the adsorption process is widely recognized as one of the most effective [2]. This is due to the fact that adsorption is inexpensive and easy to operate, and does not produce additional secondary contaminants [23,24]. Synthetic organic dyes, widely used in the textile, paper, tanning, and plastics industries, pose a particular threat to the hydrosphere. These compounds, especially cationic ones, exhibit high chemical stability, resistance to photodegradation, and barrier properties to solar radiation, which drastically limits photosynthesis in aquatic ecosystems. Furthermore, many of them are characterized by high toxicity, mutagenic, and carcinogenic effects, causing long-term effects in trophic chains [25]. In this context, biochar—defined as a dark, porous solid with a high amorphous carbon content—is a highly efficient, low-cost sorption matrix [2,26]. The mechanism of removing these pollutants in biochars is complex; it is based mainly on the strong electrostatic attraction between the adsorbent surface and cationic dye molecules, as well as on the formation of hydrogen bonds, π-π interactions, and physical penetration into the developed porous structure [26,27]. Due to the above properties, biochars are increasingly replacing expensive commercial activated carbons not only in the removal of dyes, but also heavy metals such as Pb, Cd, Cr, and As [4,28].

Buckwheat hulls are an attractive precursor for the production of biosorbents due to their specific biopolymer structure. The structural and physicochemical properties of the final product are strictly determined by the thermal process parameters, in particular the pyrolysis temperature [27,29]. As the temperature increases, intense thermal degradation, dehydration, and deoxidation of lignocellulosic components occur, resulting in an increase in the content of fixed carbon and ash, while simultaneously reducing the share of oxygen and hydrogen [29,30]. Such treatment directly stimulates the development of mesopore and micropore volume, leading to an increase in specific surface area, which translates into kinetic efficiency and adsorption capacity for cationic dyes [29]. Furthermore, the surface of buckwheat hull biochar is naturally rich in acid-base functional groups (including carboxyl, hydroxyl, and amine), which play a key role in contaminant binding [2]. These properties can be further improved by chemical modifications or the creation of composite structures, e.g., with metal–organic frameworks (MOFs), which increases the selectivity of pollutant removal from approximately 50% to even 80% [27,31]. Previous studies on the stability and reactivity of buckwheat hull biochars indicate that they can successfully compete with traditional, commercial activated carbons [31].

Taking these considerations into account, this work focuses on analyzing the effect of pyrolysis temperature on the structural, textural, and surface properties of carbon adsorbents obtained from buckwheat hulls. The primary research objective is to assess their application efficiency in the removal of model cationic dyes from the aquatic environment. The conducted analyses are consistent with the modern trend of searching for sustainable and effective industrial wastewater treatment technologies, achieving synergy between agri-food waste management and the protection of biosphere resources.

## 2. Materials and Methods

### 2.1. Reagents and Adsorbates

The standards of both cationic dyes, Rhodamine B (RhB) and Crystal Violet (CV), were obtained from Sigma-Aldrich (St. Louis, MO, USA). Table 1 presents their structures and key parameters. The other reagents (analytical-grade) used in the production and characterization of biochars, as well as in adsorption studies, were purchased from the Polish companies Chempur (Piekary Śląskie, Poland) and Stanlab (Lublin, Poland).

### 2.2. Buckwheat Husk

Buckwheat husks used as feedstock for biochar production were obtained from agricultural farms situated in central-western Poland.

The chemical composition of buckwheat husk biomass on a dry matter basis was determined in accordance with the analytical procedures of the Technical Association of the Pulp and Paper Industry (TAPPI). The following components were analyzed: (I) cellulose content using the Seifert method with an acetylacetone–dioxane mixture [32]; (II) holocellulose content by the sodium chlorite method (TAPPI T 9 wd-75); (III) pentosan content according to Tollens’ method with phloroglucinol (TAPPI T 233 cm-84); (IV) lignin content using concentrated sulfuric acid (TAPPI T 222 om-06); (V) solvent-extractable compounds determined by Soxhlet extraction (TAPPI T 204 cm-07); and (VI) ash content (TAPPI T 211 cm-86). The theoretical hemicellulose content was calculated as the difference between holocellulose and cellulose contents.

### 2.3. Preparation and Characterization of Buckwheat Husk Biochars

Buckwheat husks were oven-dried at 105 °C until a constant mass was achieved. The dried biomass was subsequently carbonized in a muffle furnace (Czylok, Jastrzębie-Zdrój, Poland) under oxygen-limited conditions with controlled operational parameters. Pyrolysis was carried out at 500, 600, and 700 °C, with a heating rate of 3 °C min^−1^ and a 1 h hold at the final temperature. After carbonization, the biochar yield was determined by the mass of the resulting material. The percentage yield was calculated using Equation (1)

(1) $$yield=\frac{w_{husk}}{w_{biochar}}\cdot 100\%$$

where *w*_biochar_ is the biochar weight (g), and *w*_husk_ is the dry buckwheat husks weight (g).

The iodine number of biochars derived from buckwheat husks was determined using a redox titration method according to the ASTM D4607-14 standard [33]. The iodine number (IN) was calculated using the following equation:

(2) $$iodine\;number=\frac{\left(V_{1}-V_{2}\right)\cdot c\cdot 126.92}{m}$$

where *V*_1_—the volume of sodium thiosulfate solution used in the blank (mL), *V*_2_—the volume of sodium thiosulfate solution used in the actual determination (mL), *c*—titer of sodium thiosulfate solution (mol/L), *m*—mass of biochar (g), and 126.92—the mass of 0.5 moles of iodine (g).

The quantitative analysis of oxygen-containing acidic and basic functional groups present on the biochar surface was conducted using the titration method. Biochar samples (0.25 ± 0.01 g) were accurately weighed and transferred separately into 250 mL Erlenmeyer flasks. Subsequently, 25 mL of one of the following solutions was added to each flask: 0.1 mol/L NaOH or 0.1 mol/L HCl. The mixtures were agitated for 24 h at approximately 180 rpm and then filtered. The obtained filtrates were titrated with 0.1 mol/L HCl (for the determination of basic groups) or 0.1 mol/L NaOH (for acidic groups) using Tashiro’s indicator. All analyses were performed in triplicate. The amount of surface functional groups (*A*_x_, mmol/g) was calculated according to the following equation:

(3) $$A_{x}=\frac{(V_{0}-V_{1})\;n_{t}\;25}{N_{1}}$$

where *V*_0_ and *V*_1_ are the volumes of HCl or NaOH solution used for the titration of the blank and the biochar sample, respectively; *n*_t_ is the concentration of the titrant (mol/L); and *N*_1_ is the mass of the biochar sample (g).

The ash content of the biochars was determined following a modified ASTM D2866 procedure. Approximately 1.0 ± 0.1 g of pre-dried biochar sample was accurately weighed and placed in a crucible, which was then introduced into a cold muffle furnace. The temperature was gradually increased to 500 °C over 30 min and held constant for 30 to 50 min. Then, the temperature was increased to 650 ± 25 °C and held for 90 min. After combustion, the crucible was removed from the furnace, cooled to room temperature in a desiccator, and weighed. The ash content was calculated using Equation (4)

(4) $$ash\;content=\frac{m_{R}-m_{0}}{m_{S}-m_{0}}\cdot 100\%$$

where *m*_0_—mass of the empty crucible (g), *m*_S_—mass of the crucible with sample (g), and *m*_R_—mass of the crucible with residue (g).

The point of zero charge (pH_PZC_) of the BHBs was evaluated using a pH drift method. For this purpose, 20 mL portions of 0.01 mol/L NaCl solution were placed in Erlenmeyer flasks, and their initial pH values were carefully adjusted to a range of 2–12 using dilute HCl or NaOH solutions of the same concentration. Subsequently, 0.05 g of biochar was added to each flask, and the suspensions were agitated for 24 h to allow the system to reach equilibrium. Afterward, the solid phase was separated by filtration, and the final pH of each solution was recorded. The pH_PZC_ was then obtained from the plot of ΔpH (defined as pH(final) minus pH(initial) versus the initial pH, as the point where the curve intersects the x-axis.

Thermogravimetric analysis (TGA) was performed using an STA 449 F5 Jupiter-QMS thermal analysis test stand (NETZSCH Group, Burlington, MA, USA). The sample (25 ± 1 mg) was heated from 30 °C to 900 °C at a rate of 10 °C/min, with a helium flow rate of 20 cm^3^/min. Instrument control, data acquisition, and data processing were carried out using Proteus Software for Thermal Analysis (version 6.1.0, NETZSCH Group, Burlington, MA, USA).

The total C, H, N, and S atom content of the biochars (wt.%) was measured using a Vario EL Cube elemental analyzer (Elementar Analysensysteme, Langenselbold, Germany). The total oxygen content was also estimated (by calculating 100% minus the sum of C%, H%, N%, and S%).

The surface morphology and elemental composition of the biochar samples were examined by scanning electron microscopy coupled with energy-dispersive spectroscopy (SEM) using a Zeiss EVO 10 microscope (Carl Zeiss Microscopy GmbH, Jena, Germany). Prior to analysis, the samples were mounted on aluminum stubs using conductive carbon tape and sputter-coated with a thin gold layer employing a Quorum Q150R Plus coater (Quorum Technologies Ltd., Laughton, UK).

### 2.4. Batch Adsorption Experiments

Batch adsorption experiments were carried out in duplicate at 23 °C as follows. A 40 mL solution of RhB and CV at an appropriate concentration was placed in Erlenmeyer flasks together with a pre-weighed amount of buckwheat husk-derived biochar (BHB). The mixtures were shaken at 200 rpm, then filtered, after which the concentrations of both dyes in the filtrate were determined. The residual dye content was determined by UV-Vis spectrophotometry (Varian Carry 3E, Palo Alto, CA, USA). The calibration curves for both dyes (1–30 μmol/L) were y = 0.0752x − 0.024 (*R*^2^ = 0.9998) for CV and y = 0.0946x + 0.0149 (*R*^2^ = 0.9995) for RhB, respectively.

The adsorption experiments investigated the effects of the adsorbent dose, solution pH, and ionic strength on dye removal, as well as adsorption kinetics and isotherms. In the adsorbent dose experiments, the dye concentration was fixed at 30 μmol/L, and the BHBs content was varied from 5 to 20 mg (from 0.125 to 0.5 g/L). The impact of the initial pH was studied over the range 3–9. The solution pH was adjusted to the desired value using 0.1 mol/L NaOH or 0.1 mol/L HCl. The concentrations of CV and RhB were both set at 30 μmol/L, while the BHB dose was fixed at 0.5 g/L. To investigate the effect of ionic strength on adsorption, the appropriate amount of sodium chloride was added to the dye solutions (*C*_0_ = 30 μmol/L, *V* = 40 mL, and *m* = 0.02 g) to yield final NaCl concentrations of 0.1, 0.5, 1.0, and 2.0 mol/L. The adsorption kinetics were studied at initial concentrations of CV and RhB of 30 μmol/L, with a 0.5 g/L adsorbent dose. Adsorption isotherms were determined over a range of dye concentrations from 5 to 70 μmol/L. The experiments were conducted in unbuffered dye solutions with a natural (original) pH of ~5.7 for CV and ~4.6 for RhB. Experiments examining how solution pH influences adsorption were an exception.

The adsorption capacities of CV and RhB on BHBs at a given time (*q*_t_, μmol/g) and in equilibrium (*q*_e_, μmol/g) were assessed according to the following equations:

(5) $$q_{t}=\frac{(C_{0}-C_{t})V}{m}$$

(6) $$q_{e}=\frac{(C_{0}-C_{e})V}{m}$$

where *C*_0_, *C*_e_, and *C*_t_ (μmol/L) are the initial CV or RhB concentration, concentration under equilibrium, and concentration at time t, respectively; *m* is the mass of BHBs (g), and *V* is the volume of the dye solution (L).

All adsorption experiments were performed in duplicate, and the average of the two results was used in all calculations.

## 3. Results and Discussion

### 3.1. The BHBs Characterization

The chemical characterization of buckwheat husks showed that the biomass consisted of 39.65% cellulose, 33.90% lignin, 22.41% pentosans, 24.36% hemicellulose, 1.77% ash, and 2.00% solvent-extractable compounds. For comparison, buckwheat husk from the Hokkaido University test field in Japan [34] consisted of cellulose (27.77 ± 0.93%), hemicellulose (6.06 ± 0.18%), and lignin (26.12 ± 1.06%). The total solids content of the raw biomass was 92.82 ± 0.39%. Buckwheat hulls from a Ukrainian processing plant [1] consisted of lignin (36.3 ± 0.1%), cellulose (27.4 ± 0.1%), hemicellulose (15.5 ± 0.2%), protein (4.8 ± 0.1%), and pectin (3.1 ± 0.1%). The ash content of the raw material was determined to be 3.6%.

The values of the main parameters characterizing the properties of the biochars obtained from buckwheat husks are listed in Table 2.

The yield of biochar depends on the raw material characteristics and carbonization conditions (temperature or residence time) [35,36,37]. In general, as the pyrolysis temperature increases, the biochar yield decreases, as observed in our work. As presented in Table 2, the yields of BHB500, BHB600, and BHB700 were calculated to be 36.3%, 35.2%, and 34.0%, respectively. The observed decrease in yield suggests that higher temperatures promote the gradual breakdown of complex (large) organic compounds in buckwheat husks into smaller molecules, which can then partially convert into volatile gases [38]. A decrease in the yield of buckwheat husk-derived biochar from 46.3% to 28.5% with increased temperature from 350 to 650 °C was reported by Zama et al. [30].

The ash content of BHBs increased with increasing pyrolysis temperature, consistent with the commonly observed trend [35,39].

With increasing pyrolysis temperature, the number of surface acidic functional groups (such as carboxyl and phenolic hydroxyl groups) decreased, while the amount of basic functional groups increased. The pH_PZC_ of the BHBs also indicates an increase in the biochar’s alkalinity. Biochars are generally basic, and their alkalinity increases with the pyrolysis temperature [35,36,37]. The increase in pH of the biochar with increasing temperature results from thermal decomposition of acidic, oxygen-containing groups during pyrolysis and from the increase in ash content, which exhibits strongly alkaline properties.

The porosity of biochar samples obtained at temperatures in the range 500–700 °C shows only small differences (iodine number (Table 2) is in the range 130–140 mg/g). It is only about 7.5%. The iodine number is a parameter used as an indicator of the porosity (microporosity) and specific surface area of materials [40]. Generally, the iodine number increases with increasing microporosity and specific surface area of the materials. Although the iodine number cannot be directly converted into a specific surface area, based on literature data [40,41,42], the obtained iodine number values should correspond to a BHB surface area of a few m^2^/g. As reported by Zama and collaborators [30], the BET surface areas of buckwheat husk-derived biochars produced at 350, 450, 550, and 650 °C were found to be 11.40, 10.72, 17.02, and 17.80 m^2^/g, respectively. The specific surface areas of buckwheat husk biochars are therefore not too high, but nevertheless within the range (~1.5–500 m^2^/g) reported for other biochars [36,37]. Usually, the surface area of biochar increases during pyrolysis due to the decomposition of hemicellulose and cellulose as well as the formation of porous structures [36,37,39]. The low surface areas of buckwheat husk biochars are probably due to inorganic materials, tars, and other products of the thermal decomposition of biomass that partially fill or block the micropores [39].

**Figure 1:**
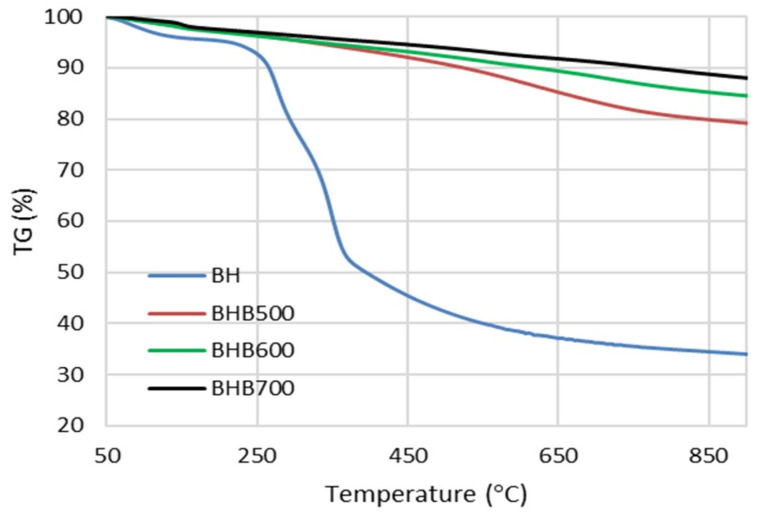
TG curves registered for biochars obtained from buckwheat husks.

For all three biochars and buckwheat husks, thermogravimetric analysis was performed. The registered TG curves are presented in Figure 1.

Their courses are similar, but for samples obtained at higher temperatures, smaller mass losses were observed. For example, at a carbonization temperature of 700 °C, it was 9.1%; at 600 °C, 11.8%; and at 500 °C, 16.6%. These differences can result from increased ash content and decreased Σ of acidic groups, as well as higher carbonization temperatures. Similar differences are evident in the temperature ranges (Table 3).

With increasing temperatures, one can observe increasing differences in mass loss values for BHB500 and BHB700 biochar samples. For deeper interpretation, the results of thermogravimetric analysis, as well as the DTG results (Table 4), are discussed.

As shown in Table 4, the tested biochars differed in their thermal stability, which increased with increasing pyrolysis temperature. The lowest thermal stability was observed for sample BHB500, which had the highest total mass loss (20.90%), while the highest thermal resistance was demonstrated by sample BHB700, for which the total mass loss was only 12.02%. Intermediate properties were observed for sample BHB600 (15.52%).

Thermal decomposition of all samples occurred in two stages. The first stage, occurring in the temperature range of 30–200 °C, was associated with dehydration and the removal of physically adsorbed water. The corresponding mass losses were relatively small and comparable for all biochars, ranging from 2.56 to 3.07%, with DTG peaks observed at temperatures of approximately 94–151 °C.

The second stage, occurring in the 400–800 °C range, corresponded to the gradual decomposition of the organic carbon structure and secondary degassing processes. Differences between the samples were observed in this region. BHB500 biochar exhibited a broad decomposition peak with a DTG peak at 615 °C and the highest mass loss at this stage (16.60%), indicating the presence of thermally less stable carbon fractions and functional groups, as well as partial carbonization resulting from the lower pyrolysis temperature. In contrast, BHB600 and BHB700 samples were characterized by the presence of two distinct DTG peaks in the second decomposition stage.

For BHB600, the peaks occurred at 553 and 721 °C, while for BHB700, they were shifted towards higher temperatures (562 and 759 °C). This phenomenon suggests the presence of more condensed and thermally resistant aromatic structures formed at higher pyrolysis temperatures. Furthermore, the smaller mass losses observed for BHB600 (7.88%) and BHB700 (5.59%) confirm the progressive increase in the degree of structural order and carbonization with increasing processing temperature.

DTA results (Table 4) further confirm these observations. Endothermic effects associated with the second decomposition step increased significantly with pyrolysis temperature, and the enthalpy values were 9.61, 38.74, and 53.72 µVs/mg for BHB500, BHB600, and BHB700 samples, respectively. Higher enthalpy values for BHB600 and especially for BHB700 indicate that the decomposition of their highly condensed carbon structures required a higher energy input, confirming their higher thermal stability.

The elemental compositions of the buckwheat husk biochars were determined, and together with calculated atomic ratios (H/C, O/C, and (O+N)/C) are listed in Table 5.

In general, increasing pyrolysis temperature increased carbon (C) content due to greater carbonization at higher temperatures. The hydrogen (H) and oxygen (O) contents decreased as the temperature increased from 500 to 700 °C, indicating that decarbonylation, decarboxylation, and dehydration occurred during pyrolysis [43]. The nitrogen (N) and sulfur (S) contents remained unchanged with pyrolysis temperature. As the pyrolysis temperature increased, the H/C atomic ratio decreased for all biochars, indicating an increase in aromaticity of BHBs. The decrease in the polarity index [(O+N)/C] and the O/C atomic ratio with increasing pyrolysis temperature suggests a reduction in surface oxygen-containing functional groups, mainly acidic (Table 2). These observations are confirmed by the mass loss values in the TG curves (Figure 1). As the pyrolysis temperature increased, the BHBs became less hydrophilic, with weaker polar groups [30]. Similar results were reported by other authors [30,31] who have studied biochars derived from buckwheat husks.

**Figure 2:**
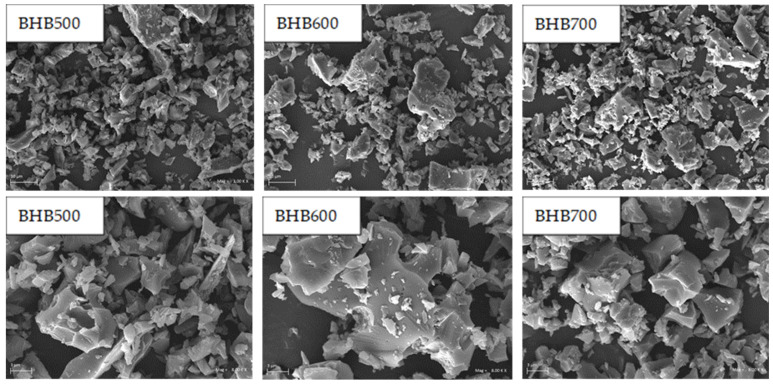
SEM images of buckwheat husk-derived biochars under different processing conditions. Scale bars: 10 µm (**top** row) and 3 µm (**bottom** row).

Figure 2 shows SEM images of biochars obtained from buckwheat husks at pyrolysis temperatures of 500, 600, and 700 °C.

All samples (Figure 2) exhibit irregular particle morphology and heterogeneous surface structures typical of biomass-derived carbon materials. The particles are characterized by rough surfaces, fractures, and cavities formed during the thermal decomposition of the lignocellulosic components and the evolution of volatile matter. The BHB500 sample displays a relatively compact structure with numerous fragmented particles, whereas BHB600 contains larger plate-like carbonaceous fragments with smoother surfaces. Further increasing the pyrolysis temperature to 700 °C resulted in a more fragmented morphology and increased surface heterogeneity. Higher-magnification images confirm the presence of pores, cracks, and surface defects in all biochars, indicating the development of porosity during the carbonization process.

### 3.2. Adsorption of Dyes on Buckwheat Husk Biochars

#### 3.2.1. Effect of BHBs Dose

**Figure 3:**
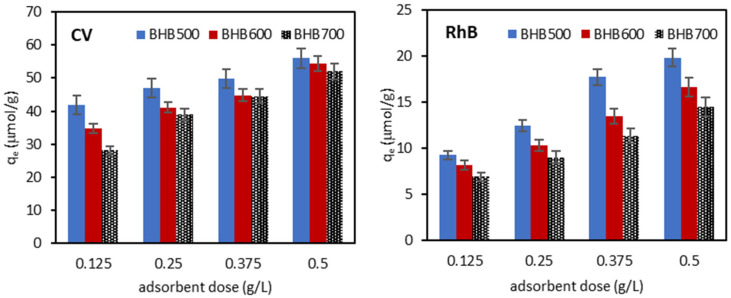
Influence of BHBs dosage on the adsorption of CV and RhB from aqueous solutions.

The effect of adsorbent amount on the adsorption of both dyes was investigated at four biochar dosages: 0.005, 0.01, 0.015, and 0.020 g. Converted to solution volume, this yielded the following doses: 0.125, 0.25, 0.375, and 0.5 g/L. The results obtained are shown in Figure 3 as a plot of *q*_e_ versus adsorbent dose.

As demonstrated, a higher adsorbent dose results in greater adsorption of the dyes. This aligns with expectations, as adding more adsorbent while maintaining a constant number of adsorbate molecules increases the number of active surface sites, thereby enhancing adsorption efficiency. Further adsorption experiments were conducted using a constant adsorbent mass of 0.02 g (equivalent to 0.5 g/L).

#### 3.2.2. Effect of Solution pH on Dye Adsorption

Solution pH is an essential parameter that influences adsorption by changing the ionic form of the adsorbate molecule and modifying the charge on the adsorbent surface. The point of zero charge (pH_PZC_) is the pH at which the adsorbent surface has zero charge. In an environment with a pH below the pH_PZC_, a positive charge accumulates on the surface; conversely, at a pH above the pH_PZC_, the adsorbent surface becomes negatively charged. Experimentally determined pH_PZC_ values for BHB500, BHB600, and BHB700 were found to be 7.30, 7.55, and 7.80, respectively. Therefore, in acidic and neutral environments, the surface of BHBs is positively charged, and in alkaline environments, it is negatively charged.

Crystal Violet is a cationic triphenylmethane dye that contains three tertiary amino groups. These undergo protonation or deprotonation depending on the solution’s pH. Consequently, the dye can exist in the following forms: (i) a trication when all three nitrogen atoms are protonated (at pH below 1); (ii) a dication (at pH ~1.8–2.6); and (iii) a triphenylmethane monocation (at pH ~3.0–9.4) [44,45]. Studies on the effect of pH on dye adsorption onto BHBs were therefore conducted in the pH range of 3 to 9, where CV exhibits a stable, characteristic violet color. In a strongly basic environment (pH > 9.4), CV begins to lose its color due to the delocalization of its aromatic rings by OH^−^ ions. This results in the formation of the colorless triphenylmethanol (carbinol) form of the dye [45].

Rhodamine B is a xanthene dye that can exist in either a cationic or zwitterionic form in an aqueous solution. At low pH (acidic conditions), RhB predominantly exists in a highly protonated form in which both nitrogen atoms in its xanthene ring are protonated. This results in a positively charged dicationic species. This form is stabilized by the abundance of protons in the acidic environment. In contrast, deprotonation occurs as the pH increases and the medium becomes basic. The pKa of RhB is approximately 3.7. This means that at pH < 3.7 (acidic conditions), the nitrogen atoms in the xanthene ring and the carboxyl group are protonated, and the dye predominantly exists in a cationic form. As the pH exceeds 3.7, the carboxyl group deprotonates, rendering RhB zwitterionic [46].

**Figure 4:**
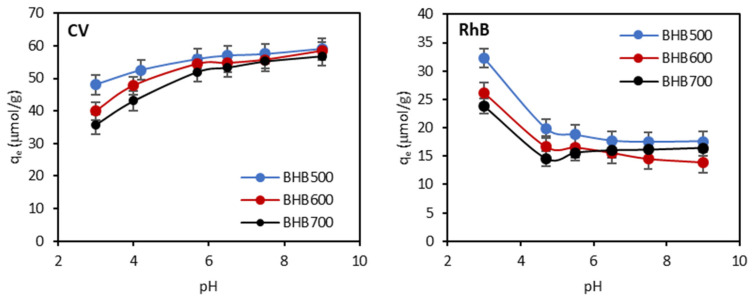
Effect of solution pH on the adsorption of CV and RhB on BHBs.

The effect of solution pH on the adsorption of CV and RhB onto buckwheat husk-derived biochars is shown in Figure 4. The results clearly demonstrate that solution pH plays a crucial role in the adsorption of CV and RhB from the aqueous phase, although the effects on adsorption differ between the dyes.

As shown in Figure 4, CV adsorption was lowest in an acidic environment for all three biochars (under these conditions, the CV molecule is a monocation, and the biochar has a positive surface charge). Next, the adsorption capacities (*q*_e_) increased with increasing pH from 3 to 9. In the alkaline solution, the dye molecule remains a single-charged cation. However, the adsorbent surface becomes negatively charged at pH values above pH_PZC_. A comparison of CV adsorption at pH 3 and pH 9 shows an increase in the adsorption capacities of BHB500, BHB600, and BHB700 by 18.6%, 30.7%, and 35%, respectively. A similar effect of pH on CV adsorption from water has been reported for other biochars derived from calves’ horn core [47], chinar tree leaf powder [48], sugarcane bagasse waste [49,50], rice straw [50], palm kernel shell [51], and azedarach tree seeds [52].

In contrast, RhB was most effectively adsorbed in an acidic environment (pH = 3). The adsorption efficiency of this dye decreased as the pH increased from 3 to around 5. No further changes in adsorption occurred with increasing solution pH. A similar trend was observed during the adsorption of RhB onto goat manure [53], cotton straw [54], or tomato stem-derived [42] biochars.

These results clearly indicate that the solution pH plays a key role in the adsorption of CV and RhB from the aqueous phase, although the effects on these dyes differ. This is due to the differences in the physicochemical properties of these dyes and their distinct adsorption mechanisms on BHB surfaces.

The adsorption of dyes on various adsorbents, including biochars, is a complex process involving various mechanisms such as pore filling, surface complexation, ion exchange, hydrogen bonding as well as electrostatic and π–π interactions [35,36,37,39,55,56].

The lowest adsorption of CV in an acidic environment is due to the strong protonation of the biochar surface’s functional groups. Under these conditions, positive surface charges predominate, leading to electrostatic repulsion between the cationic dye (CV) and the adsorbent’s positively charged surface. This effect may be further enhanced by increased H^+^ ion concentrations, as these ions compete with the dye cations for active adsorption sites [38,45,49,50,52]. As pH increases, the adsorbent surface gradually becomes deprotonated, leading to a negative charge. This strengthens the attractive electrostatic interactions between the dye molecule and the adsorbent surface. At the same time, the contribution of other interactions, such as hydrogen bonding and π–π interactions between the dye and the adsorbent, increases [37]. Consequently, a marked increase in adsorption capacity is observed as the pH increases, reaching a maximum in neutral and basic environments. The behavior of CV as a function of solution pH suggests that the adsorption mechanism is dominated by electrostatic interactions. However, the CV adsorption process may also be supported by other mechanisms [37].

Conversely, RhB’s behavior in response to changes in solution pH suggests that the electrostatic attraction mechanism is of secondary importance in this case. As RhB is a cationic dye, if adsorption were primarily due to electrostatic interactions, it would adsorb most efficiently under neutral/basic conditions, as with CV, and least efficiently in a strongly acidic environment (due to repulsive electrostatic interactions between the cationic form of RhB and the positively charged surface of BHB). However, the best adsorption efficiency was observed precisely at the lowest pH (3.0). It was reported [42,54,57,58,59,60] that the adsorption of RhB on biochar-based adsorbents involves multiple mechanisms simultaneously, including hydrophobic, electrostatic, π–π, and hydrogen bonding interactions. In the case of RhB adsorption on BHBs, hydrophobic and π–π interactions, as well as hydrogen bonding, appear to play a key role in the adsorption mechanism.

#### 3.2.3. Effect of Ionic Strength on Dyes Adsorption

**Figure 5:**
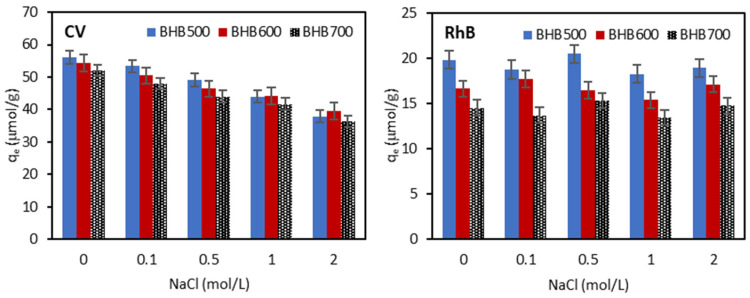
Effect of solution ionic strength on the adsorption of CV and RhB on BHBs.

Figure 5 presents the influence of solution ionic strength on the adsorption of CV and RhB on BHBs.

The adsorption of CV from NaCl solutions decreased as the concentration of salt and, consequently, the ionic strength of the solution increased. An increase in NaCl concentration from 0 mol/L (pure water) to 2 mol/L decreased the adsorption capacity by approximately 30.1%, 24.9%, and 26.2% for BHB500, BHB600, and BHB700, respectively. This may be because Na^+^ ions occupy the available surface-active sites on biochar, reducing their negative charge and weakening the electrostatic attraction between the dye and these sites. Consequently, an increase in ionic strength negatively affects adsorption efficiency. Similar results were obtained by other researchers [49,51,52]. On the other hand, Nouioua et al. [47] reported that solution ionic strength has a minimal effect on CV adsorption onto horn-derived biochar. An increase in NaCl concentration from 0.05 to 1 mol/L results in a slight reduction in adsorption of about 2.3%.

The results for RhB (Figure 5) show that adsorption from solutions with different salt concentrations was approximately constant. The coefficient of variation values calculated for all five solutions were 4.7% for BHB500, 5.2% for BHB600, and 5.3% for BHB700. Low values of this parameter (~5%) indicate low variability and suggest that the slight adsorption fluctuations observed are due to measurement errors rather than real changes in adsorption efficiency. The effect of coexisting cations on RhB adsorption onto modified biochar from coconut shell was studied by Li and collaborators [57]. They found that RhB adsorption increased slightly as the concentrations of Na^+^ and K^+^ ions in the solution increased. However, it decreased significantly in the presence of Mg^2+^ and Ca^2+^. On the other hand, the adsorption of RhB onto tomato stems-derived biochar was independent of the solution’s ionic strength [42].

In summary, the results showed that the presence and concentration of inorganic salts (ionic strength) in the solution reduced the efficiency of CV adsorption on BHBs. However, this did not affect RhB adsorption. These findings indirectly support our earlier observations (Section 3.2.2) that CV adsorption primarily occurs via attractive electrostatic interactions. In the case of RhB, which is insensitive to the presence of other ions, electrostatic interactions play a less significant role.

#### 3.2.4. Effects of Contact Time and Adsorption Kinetics

**Figure 6:**
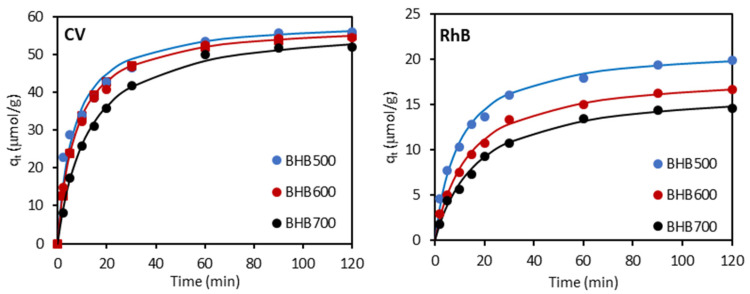
Effect of contact time on CV and RhB adsorption on the BHBs (the lines represent the model fitting of the PSO kinetic model).

The effect of contact time was studied for an initial dye concentration of 30 μmol/L and a BHB dose of 0.5 g/L. The obtained results are listed in Figure 6.

Adsorption of CV and RhB occurs very rapidly at first, with approximately 70–80% of their final amounts adsorbed within the first 20 min. It then slows down and reaches equilibrium after about 60 min. As differences in the adsorption rates of CV and RhB, as well as between individual biochars, are not immediately apparent, appropriate kinetic models were used to describe the obtained results. These were the pseudo-first-order (PFO), pseudo-second-order (PSO), and Avrami models, which are described by the following equations [61,62]:

(7) $$q_{t}=q_{e}(1-\exp(-k_{1}t))$$

(8) $$q_{t}=\frac{k_{2}tq_{e}^{2}}{1+k_{2}q_{e}}$$

(9) $$q_{t}=q_{e}[1-exp{\left(-k_{A}t\right)}^{n_{A}}]$$

where *k*_1_ is the PFO adsorption rate constant (1/min), *k*_2_ is the PSO model rate constant (g/μmol∙min), *k*_A_ is the Avrami rate constant (1/min), and *n*_A_ is the dimensionless fractional exponent of the Avrami model.

The constants for these kinetic models were obtained by nonlinear least-squares analysis in OriginPro 8.0. (OriginLab Corporation, Northampton, MA, USA). To assess the validity of the models, the coefficient of determination (Equation (10)) and root-mean-square error (Equation (11)) values were used.

(10) $$R^{2}=\frac{\sum_{i=1}^{n}{\left(q_{e\left(cal\right)}-\bar{q_{e(exp)}}\right)}^{2}}{\sum_{i=1}^{n}{\left(q_{e\left(cal\right)}-\bar{q_{e(exp)}}\right)}^{2}+\sum_{i=1}^{n}{\left(q_{e\left(cal\right)}-q_{e\left(exp\right)}\right)}^{2}}$$

(11) $$RMSE=\sqrt{\frac{1}{n}\sum_{i=1}^{n}{\left(q_{e\left(exp\right)}-q_{e\left(cal\right)}\right)}^{2}}$$

In these equations, the parameters *q*_e(cal)_ and *q*_e(exp)_ denote the adsorption capacities calculated from the theoretical model and obtained experimentally, respectively. A higher R^2^ and lower RMSE indicate that the theoretical model fits the experimental data better. The results are listed in Table 6.

Analysis of the *R*^2^ and RMSE values presented in Table 6 shows that the adsorption kinetics of CV and RhB on BHBs follow the pseudo-second-order model. This is evident from the lowest RMSE values (≤1.316) and the highest *R*^2^ values (≥0.998) obtained with this model. The Avrami model also showed good agreement, whereas the PFO model exhibited the poorest fit to the experimental data. The values of the constant adsorption rates in the PSO model increase in the order BHB700 < BHB600 < BHB500. Therefore, both dyes were adsorbed most quickly on BHB500 and most slowly on BHB700.

This is also confirmed by the *t*_1/2_ values (the time required to reach 50% of the adsorption capacity), which were calculated using the following equation:

(12) $$t_{1/2}=\frac{1}{q_{e(cal)}\cdot k_{2}}$$

It was found that 50% of the CV adsorption capacity was achieved after 6.4, 7.2, and 12.7 min for BHB500, BHB600, and BHB700, respectively. For RhB, the adsorption half-times were 9.6 min for BHB500, 13.2 min for BHB600, and 16.7 min for BHB700.

Given the changes in the physicochemical properties of biochars at different pyrolysis temperatures (Table 2), it seems that the surface chemistry of these materials is crucial for adsorption. The specific surface area of BHBs, estimated from the iodine number and literature data [30], is low, around a dozen m^2^/g. The porous structure of these BHBs (including the mesopore volume, which plays the most important role in the adsorption rate) is therefore poorly developed. The porosity of these three BHBs does not differ significantly, as indicated by similar iodine numbers (130–140 mg/g). However, as described in Section 3.1, the pyrolysis temperature significantly influenced the resulting biochars’ chemical properties, with acid-base properties being the most noticeable parameter. When the kinetic parameters presented in Table 6 are compared with the properties of the BHBs (Table 2), a tendency can be observed: the adsorption rate of both dyes on the BHBs decreases as the biochars become more alkaline.

This phenomenon is also supported by the Avrami model results. The *k*_A_ rate constants determined for both dyes followed the same order (BHB700 < BHB600 < BHB500). The Avrami model can be useful not only for determining and comparing adsorption rates but also for identifying the adsorption mechanism. The adsorption mechanism involving mass transfer proceeds in three main stages: (i) film diffusion, (ii) pore diffusion (intraparticle diffusion), and (iii) adsorbate-adsorbent interactions (physicochemical adsorption of adsorbates at active sites on the adsorbent) [62]. The overall rate of the process is controlled by the slowest of these stages. The Avrami kinetic constant (*n*_A_) helps identify the adsorption mechanism and dimensionality of the entire process. If the value is different from 1, this indicates that the process is complex and that the adsorption rate is affected by more than one of the aforementioned stages. As stage (iii) occurs very rapidly, the rate-limiting stage of adsorption is film diffusion, intraparticle diffusion, or both. The *n*_A_ values obtained (0.599–0.876 for CV and 0.549–0.772 for RhB) appear to support this hypothesis.

To better identify the rate-limiting step in the adsorption of CV and RhB onto BHBs, the Weber–Morris kinetic diffusion model (also known as the intra-particle diffusion model) was applied [61]

(13) $$q_{t}=k_{i}t^{0.5}+C_{i}$$

where *k*_i_ (μmol/g⋅min^−0.5^) and *C*_i_ (μmol/g) are the Weber–Morris model constant.

**Figure 7:**
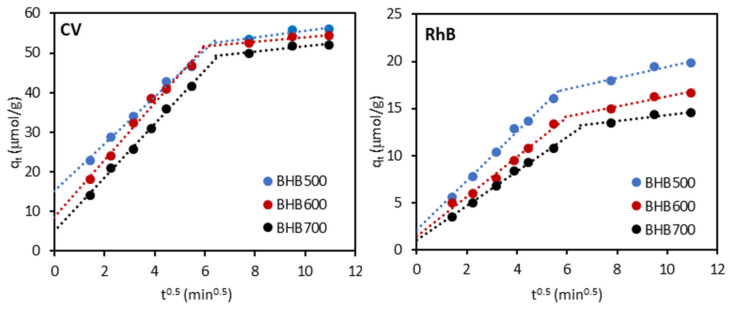
The Weber–Morris plots for the adsorption of CV and RhB on buckwheat husk biochars.

The Webber-Morris plot (*q*_t_ vs. *t*^0.5^) can be used to identify the adsorption mechanism and the rate-limiting step of the process based on the following assumptions: (i) if the *q*_t_ = f(*t*^0.5^) plot is linear and passes through the origin, then adsorption occurs only due to intra-particle diffusion; (ii) if the *q*_t_ = f(*t*^0.5^) plot is non-linear (a broken line on the graph), then several processes are involved in the adsorption; (iii) if the *q*_t_ = f(*t*^0.5^) plot is non-linear and does not pass through the origin, then adsorption is complex (its rate is affected by more than one stage), and the adsorption rate is not determined by intra-particle diffusion. The Weber–Morris plots for CV and RhB are presented in Figure 7. As can be seen, the plots of *q*_t_ vs. *t*^0.5^ are non-linear (two distinct stages can be identified), and none of the curves pass through the origin. This suggests that the adsorption rate of both dyes onto BHBs is controlled by both film diffusion and intraparticle diffusion. Furthermore, this indicates that pore diffusion is not the main rate-limiting step, and thus, adsorption kinetics is controlled primarily by film diffusion.

#### 3.2.5. Adsorption Isotherms

**Figure 8:**
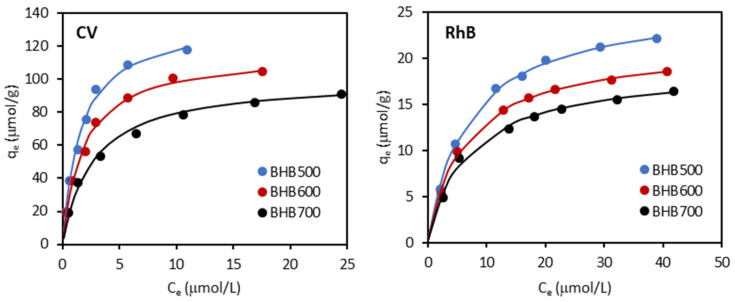
The CV and RhB dyes adsorption isotherms onto buckwheat husk-derived biochars from aqueous solutions.

The CV and RhB adsorption isotherms onto BHBs from aqueous solutions are given in Figure 8.

The Langmuir, Freundlich, and Temkin models [63] were used to characterize the experimental isotherm data. These isotherms can be represented as follows:

(14) $$q_{e}=\frac{q_{mL}K_{L}C_{e}}{1+K_{L}C_{e}}$$

(15) $$q_{e}=K_{F}C_{e}^{1/n}$$

(16) $$q_{e}=\frac{RT}{b_{T}}ln(A_{T}C_{e})$$

where *q*_m_ (µmol/g) and *K*_L_ (L/µmol) are the monolayer adsorption capacity and the Langmuir constant, respectively; *K*_F_ $\left({\left(\mu mol/g)(L/\mu mol\right)}^{1/n_{F}}\right)$ and *n*_F_ (dimensionless) are the Freundlich relative adsorption capacity and heterogeneity factor, respectively; *A*_T_ (L/g) and *b*_T_ (kJ/mol) are the Temkin isotherm constants, *R* is the gas constant (8.314 J/mol·K), and *T* is the temperature in Kelvin (296.15 K).

The isotherm constants were determined through a nonlinear least-squares approach (OriginPro 8.0). As in the kinetic studies, *R*^2^ and RMSE were used to evaluate the goodness-of-fit of the models to the experimental data. The results are listed in Table 7.

In general, all three isotherm equations described the adsorption of CV and RhB onto GHBs quite well. A more detailed analysis of the obtained *R*^2^ and RMSE values shows that the degree of fit can be ranked as follows: Freundlich < Temkin < Langmuir. The Langmuir model produced the highest R^2^ values and the lowest RMSE values, suggesting that adsorption of both dyes on the studied BHBs likely occurs via chemisorption in a monolayer on the homogeneous surface of the adsorbent. These conclusions follow from the theoretical assumptions of the Langmuir isotherm model [63]. However, it should be noted that this is not conclusive, particularly given the fairly good fit of the Temkin and Freundlich models and the lack of clear differences between the three models.

Based on the Langmuir isotherm, and more specifically on the determined *K*_L_ parameter, one can calculate the Gibbs free energy of change (∆*G*°) and separation factor (*R*_L_), which provide information regarding the type and nature of adsorption. The ∆*G*° and *R*_L_ were calculated using the following equations [63,64]:

(17) $$∆G^{{}^{\circ}}=-RT\;ln(55.5K_{L})$$

(18) $$R_{L}=\frac{1}{1+K_{L}C_{0}}$$

where R is the universal gas constant (8.314 J/mol·K), T is the temperature expressed in Kelvin (296.15), *K*_L_ is the Langmuir isotherm constant (L/μmol), and *C*_0_ is the initial dye concentration (μmol/L). The calculated values for both of these parameters are given in Table 8.

Negative Δ*G*° values indicate a favorable and spontaneous adsorption process. The *R*_L_ values range from 0 to 1 (0 < *R*_L_ < 1), indicating the favorable nature of the adsorption process.

Both dyes were adsorbed most effectively onto BHB500 and least effectively onto BHB700 (BHB700 < BHB600 < BHB500). Given the relatively less developed porous structure of the BHBs, their adsorption capacity is likely due to their surface chemistry. As discussed earlier, the most obvious changes associated with pyrolysis temperature are the acid-base properties of these biochars. Thus, the adsorption of both dyes decreased as the surface alkalinity of the BHBs increased. In other words, a more acidic surface promotes the adsorption of both dyes. This may be due to the presence of a large number of acidic, oxygen-containing functional groups, which favor electrostatic attraction and π–π interactions with the dye molecules [65]. As noted in Section 3.2.2, the adsorption mechanism of CV is dominated by electrostatic adsorbate-adsorbent interactions, whereas the hydrophobic and π–π interactions, as well as H-bonding, play a key role in the RhB adsorption.

#### 3.2.6. Comparison of BHBs with Other Adsorbents

Table 9 presents a comparison of the adsorption capacities of various adsorbents for the removal of CV and RhB from water. Although this comparison is not entirely conclusive, as it does not account for different experimental conditions, such as the range of adsorbate concentration, temperature, or pH, it nevertheless provides some insight into the potential of BHBs despite its limitations.

**Figure 9:**
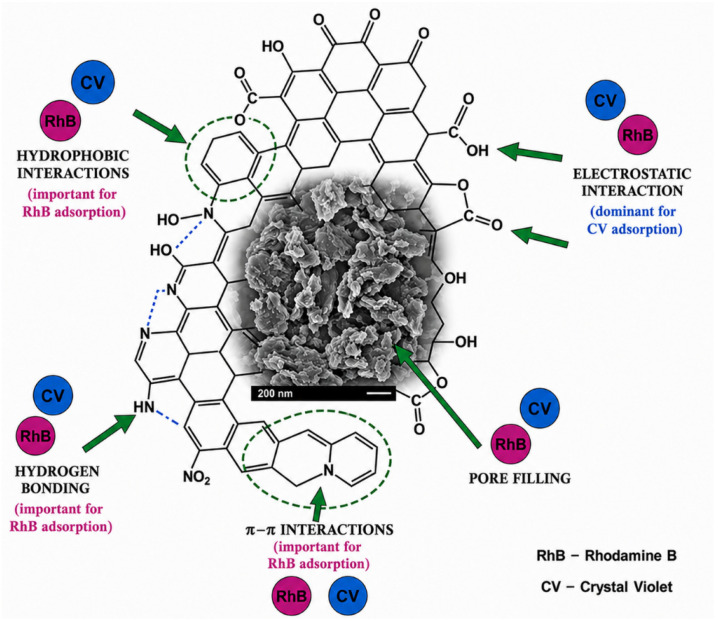
Schematic illustration of the possible adsorption mechanisms of Crystal Violet and Rhodamine B on buckwheat husk biochars.

To standardize the data, the adsorption capacities of BHBs listed in Table 9 (Langmuir constants *q*_m_) were converted to milligrams per gram. The table mainly lists the adsorption capacities of biochar-derived materials. The exceptions are studies [66,67,68,69], which are among the few in the literature describing the simultaneous adsorption of CV and RhB. The values show that the adsorption capacities of the buckwheat husk biochars used in this study are comparable to those of biochars produced from other precursors. It can also be observed that CV was adsorbed 2–3 times more effectively than RhB on each of the three BHBs. A similar trend was reported elsewhere for the adsorption of both of these dyes on non-modified and NaOH-activated Aerva javanica leaf powder [66], modified banyan aerial roots [67], and poly(styrene-co-methacrylic acid)-coated magnetite nanoparticles [68]. On the other hand, Koyuncu and Kul [69] reported opposite results—a better adsorption of RhB (344.8 mg/g) than CV (212.7 mg/g) on lichen-derived activated carbon. Therefore, more effective adsorption of CV than RhB is not the general rule, but depends on the specific properties of the adsorbent used. The greater adsorption of CV than RhB observed in this study may be due to the fact that the two dyes adsorb onto BHBs via different mechanisms. This suggests that electrostatic interactions, which are dominant in CV adsorption, are favored more in the dye-biochar system than the hydrophobic, π–π, and H-bonding interactions responsible for RhB adsorption. The possible adsorption pathways are schematically summarized in Figure 9.

## 4. Conclusions

This paper investigated the efficacy of biochars derived from buckwheat husks produced at 500, 600, and 700 °C as adsorbents for the removal of cationic dyes (CV and RhB) from aqueous solutions. Various properties of the resulting biochars were dependent on the carbonization temperature to varying degrees. Increasing the temperature caused only a slight increase in the iodine number value characterizing the porous structure, but significant changes in the surface chemistry were observed. These included a large decrease in the number of acid groups, a large mass loss in thermogravimetry (TG), and a large decrease in the O/C ratio in elemental analysis. However, the content of basic groups, ash, and pH_PZC_ showed an increase. All these differences in the biochar properties significantly influenced the adsorption kinetics and capacity of both cationic dyes. It was observed that the adsorption of both dyes was pH-dependent. The concentration of inorganic salts (ionic strength) in solution decreased the adsorption of CV and did not affect the adsorption of RhB onto the BHBs studied. The adsorption process followed a pseudo-second-order model and was primarily controlled by film diffusion. Under equilibrium conditions, the adsorption of dyes on BHBs was well described by the Langmuir, the Freundlich, and the Temkin isotherm models, with the Langmuir equation showing a slight advantage. The highest adsorption capacity was observed for BHB500, while the lowest capacity was observed for biochar obtained at a higher pyrolysis temperature (BHB700). The adsorption capacities of the BHBs for CV and RhB (BHB700 < BHB600 < BHB500) decreased as the pyrolysis temperature and the surface alkalinity of the biochars increased. The results of the study demonstrate the potential of buckwheat husk biochar as an effective adsorbent for the removal of cationic dyes from aqueous solutions. They provide theoretical support and practical guidance on utilizing agricultural waste resources to develop environmentally friendly materials.

## Figures and Tables

**Table 1:** Properties of Crystal Violet (CV) and Rhodamine B (RhB) dyes.

	Crystal Violet (CV)	Rhodamine B (RhB)
Molecular weight	407.98 g/mol	479.02 g/mol
Formula	C_25_H_30_N_3_Cl	C_28_H_31_ClN_2_O_3_
Structure	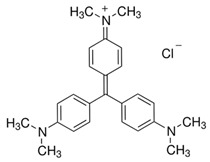	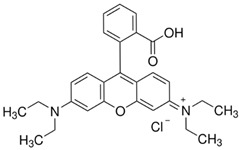
CAS number	548-62-9	81-88-9
Color index	42,555	45,170
λ max	582 nm	552 nm

**Table 2:** Characteristics of buckwheat husk-derived biochars.

Sample	Yield (%)	Ash (%)	Iodine Number (mg/g)	Σ of Acidic Groups (mmol/g)	Σ of Basic Groups (mmol/g)	pH_PZC_
BHB500	36.3	3.37	130	0.29	0.64	7.30
BHB600	35.2	3.64	135	0.14	0.79	7.55
BHB700	34.0	3.86	140	0.08	0.84	7.80

**Table 3:** Mass-loss values for the chosen temperature ranges.

Sample	Mass Loss (%) in Chosen Temperature Ranges			
	30–200 °C	200–400 °C	400–600°C	600–800 °C
Raw material—BH	4.8	45.7	10.8	3.7
BHB500	2.7	4.0	6.1	6.5
BHB600	3.0	3.2	3.5	4.2
BHB700	2.6	2.3	2.6	2.9

**Table 4:** DTG analysis of buckwheat husk biochar.

Sample	1st Mass Loss [%]	DTG Peak at 1st Mass Loss [°C]	2nd Mass Loss [%]	DTG Peak at 2nd Mass Loss [°C]	DTA Endothermic Peak Enthalpy Corresponding with 2nd Mass Loss [µVs/mg]	Total Mass Loss [%]
BHB500	2.76	94	16.60	615	9.61	20.90
BHB600	3.07	99 137	7.88	553 721	38.74	15.52
BHB700	2.56	97 151	5.59	562 759	53.72	12.02

1st stage 30–200 °C. 2nd stage 400–800 °C.

**Table 5:** CHNS elemental analysis of buckwheat husk biochars.

Sample	Atom Content in Percentage (wt.%)					Atomic Ratio		
	C	H	N	S	O *	H/C	(O+N)/C	O/C
BHB500	71.2	4.2	1.2	0.15	23.3	0.71	0.26	0.24
BHB600	76.3	3.1	1.1	0.11	19.4	0.49	0.20	0.19
BHB700	82.3	1.9	1.2	0.14	14.5	0.28	0.14	0.13

* Calculated value (O% = 100% − (ΣC% + H% + N% + S%)).

**Table 6:** Adsorption kinetic model parameters for the adsorption of CV and RhB dyes on buckwheat husk-derived biochars.

CV	Buckwheat Husk Biochar		
	BHB500	BHB600	BHB700
*q*_e(exp)_ (μmol/g)	56.01	54.41	52.02
PFO model			
*q*_e(cal)_ (μmol/g)	42.21	42.92	50.13
*k*_1_ (1/min)	5.326 × 10^−2^	5.585 × 10^−2^	5.655 × 10^−2^
*R* ^2^	0.958	0.947	0.942
RMSE	14.82	11.93	2.880
PSO model			
*q*_e(cal)_ (mmol/g)	59.02	55.58	52.93
*k*_2_ (g/μmol∙min)	2.677 × 10^−3^	2.398 × 10^−3^	1.472 × 10^−3^
*R* ^2^	0.998	0.999	0.999
RMSE	1.316	1.106	0.796
Avrami model			
*n*_A_	0.599	0.731	0.876
*k*_A_ (1/min)	0.278	0.182	0.101
*R* ^2^	0.975	0.990	0.992
RMSE	7.836	5.360	3.348
**RhB**			
*q*_e(exp)_ (μmol/g)	19.83	16.66	14.55
PFO model			
*q*_e(cal)_ (μmol/g)	15.12	13.98	10.42
*k*_1_ (1/min)	3.953 × 10^−2^	3.886 × 10^−2^	5.905 × 10^−2^
*R* ^2^	0.976	0.969	0.948
RMSE	4.860	2.841	2.921
PSO model			
*q*_e(cal)_ (mmol/g)	21.02	17.78	15.08
*k*_2_ (g/μmol∙min)	4.888 × 10^−3^	4.125 × 10^−3^	3.579 × 10^−3^
*R* ^2^	0.998	0.999	0.998
RMSE	0.449	0.359	0.388
Avrami model			
*n*_A_	0.670	0.772	0.549
*k*_A_ (1/min)	0.173	0.115	0.086
*R* ^2^	0.994	0.986	0.971
RMSE	2.283	1.682	5.503

**Table 7:** Adsorption isotherm model parameters for the adsorption of CV and RhB dyes on buckwheat husk-derived biochars.

CV	Buckwheat Husk Biochar		
	BHB500	BHB600	BHB700
Langmuir model			
*q*_m_ (μmol/g)	137.5	116.7	100.5
*K*_L_ (L/μmol)	0.634	0.552	0.389
*R* ^2^	0.998	0.999	0.998
RMSE	3.160	2.334	2.672
Freundlich model			
*K*_F_ $\left({\left(\mu mol/g)(L/\mu mol\right)}^{1/n_{F}}\right)$	46.56	37.92	30.05
1*/*$n_{F}$	0.452	0.447	0.402
*R* ^2^	0.939	0.952	0.960
RMSE	10.06	11.75	6.980
Temkin model			
*b*_T_ (kJ/mol)	0.085	0.109	0.135
*A*_T_ (L/g)	6.991	6.187	5.372
*R* ^2^	0.984	0.982	0.988
RMSE	4.020	4.350	1.323
**RhB**			
*q*_e(exp)_ (μmol/g)			
Langmuir model			
*q*_m_ (μmol/g)	26.53	21.62	19.53
*K*_L_ (L/μmol)	0.150	0.150	0.149
*R* ^2^	0.999	0.999	0.998
RMSE	0.231	0.271	0.372
Freundlich model			
*K*_F_ $\left({\left(\mu mol/g)(L/\mu mol\right)}^{1/n_{F}}\right)$	4.871	4.458	4.002
1*/*$n_{F}$	0.487	0.439	0.418
*R* ^2^	0.954	0.942	0.930
RMSE	1.786	1.459	1.120
Temkin model			
*b*_T_ (kJ/mol)	0.428	0.549	0.623
*A*_T_ (L/g)	1.362	1.608	1.589
*R* ^2^	0.992	0.982	0.995
RMSE	1.429	0.589	0.428

**Table 8:** The Gibbs free energy change (Δ*G*°) as well as the minimum and maximum values of the separation factors (*R*_L_) for the adsorption of CV and RhB on BHBs.

Sample	Δ*G*° (kJ/mol)	*R*_L_		Δ*G*° (kJ/mol)	*R*_L_	
		Min.	Max.		Min.	Max.
	CV			RhB		
BHB500	−8.767	0.022	0.136	−5.098	0.123	0.583
BHB600	−8.404	0.025	0.154	−5.269	0.114	0.563
BHB700	−7.522	0.035	0.207	−5.123	0.121	0.579

**Table 9:** Comparison of CV and RhB dyes adsorption on various biochars (BCs).

Adsorbent	CV (mg/g)	RhB (mg/g)	Ref.
BHB500	56.10	13.24	This study
BHB600	47.61	10.79	This study
BHB700	41.00	9.74	This study
*Aerva javanica* leaf powder	35.60	8.580	[66]
NaOH-activated *Aerva javanica* leaf	48.75	1.500	[66]
Modified banyan aerial roots	456.6	115.2	[67]
Poly(St-co-MAA) particles (MN_2_-P)	416.7	69.54	[68]
Lichen-derived activated carbon	218.8	344.8	[69]
BC from *Canarium schweinfurthii* stone	15.39	-	[70]
BC from palm kernel shell	24.45	-	[51]
BC from Chinar tree leaf powder	30.10	-	[48]
BC from *Melia azedarach* seeds (B700)	63.41	-	[52]
BC from sugarcane bagasse (T-BC)	99.50	-	[49]
BC from calves’ horn cores	106.1	-	[47]
BC from *Melia azedarach* seeds (HB700)	151.8	-	[52]
BC from rice straw	161.3	-	[50]
BC from sodium alginate	203.8	-	[38]
BC from sugarcane bagasse	476.2	-	[50]
BC from *Pongamia glabra* seed cover	-	0.687	[71]
BC from tomato stem (BC700)	-	4.483	[42]
BC from sugarcane bagasse (BC)	-	4.572	[65]
BC from coconut shell	-	7.840	[57]
BC from tomato stem (BC400)	-	8.887	[42]
BC from sugarcane bagasse (BCN)	-	9.756	[65]
BC from olive stones	-	11.82	[58]
BC from sugarcane bagasse (BCH)	-	16.94	[65]
BC from *Spartina alterniflora*	-	28.15	[72]
BC from goat manure	-	110.1	[53]
BC from pine nut shell:	-	110.7	[73]
BC from cotton straw	-	117.8	[54]
BC from groundnut husk	-	182.2	[60]
BC from coffee shell	-	193.5	[59]

## Data Availability

The original contributions presented in this study are included in the article. Further inquiries can be directed to the corresponding author.
